# Evaluating preference weights for the Asthma Symptom Utility Index (ASUI) across countries

**DOI:** 10.1186/1477-7525-4-51

**Published:** 2006-08-15

**Authors:** Emuella M Flood, Erwin De Cock, Ann-Christin Mörk, Dennis A Revicki

**Affiliations:** 1 Center for Health Outcomes Research, United BioSource Corporation, Bethesda, MD, United States, London, UK; 2 Formerly of AstraZeneca, Lund, Sweden

## Abstract

**Background:**

The Asthma Symptom Utility Index (ASUI) is a preference-based outcome measure used in US clinical trials and cost-effectiveness studies for asthma. This study evaluated ASUI preference weights in Europe to determine whether the multi-attribute utility function, based on preferences from a US population, is generalizable across countries.

**Methods:**

Data were collected from ninety asthma patients from Italy, France, and the United Kingdom using the Asthma Control Questionnaire, the Asthma Quality of Life Questionnaire, and the ASUI. Subjects rated their preferences for 10 asthma health states using a visual analogue scale (VAS) and a standard gamble (SG) interview.

**Results:**

All multi-symptom states showed statistically significant differences (p < 0.001) between countries in mean VAS scores. Mean SG utility scores between the US and France and the US and Italy demonstrated statistically significant differences (p < 0.001) for three states: severe wheeze; moderate cough and wheeze; and moderate cough and dyspnea. Because of these differences, the multi-attribute utility functions derived within countries were somewhat different. Despite these differences, country-specific algorithms captured a similar rank ordering of patients by disease severity, were strongly correlated (r = 0.971 to 0.995), and demonstrated similar relationships with symptom and AQLQ scores.

**Conclusion:**

Results of this study suggest that the ASUI may be a complementary patient-reported outcome for clinical studies and may be useful for applications in cost-effectiveness studies comparing different asthma treatments.

## Background

Patient-reported outcomes, such as patient perceptions of symptom frequency and severity and their health-related quality of life (HRQL) are important for clinical management and for evaluating new treatments for asthma [1]. These patient based outcomes have been used to evaluate pharmacologic and behavioral interventions in asthma [2-5]. Evaluation of the cost-effectiveness of new treatments requires careful collection of medical costs and assessment of relevant and clinically meaningful outcomes from the patient's perspective. While symptom-free days [6] and quality-adjusted life years can capture overall effectiveness, these measures may not be sensitive enough to differentiate among different active treatments for asthma [7].

The Asthma Symptom Utility Index (ASUI) is a preference-based outcome measure that can be used in clinical trials and cost-effectiveness studies for asthma [7]. It is an 11-item instrument designed to assess the frequency and severity of four asthma symptoms (cough, wheeze, dyspnea, and awaken at night) and side effects, weighted according to patient preferences. Scoring of the ASUI is based on a multi-attribute utility function, which uses utilities as the underlying weighting metric. Utilities represent patients' preferences for different health outcomes under conditions of uncertainty [8,9]. For the ASUI, utilities for different asthma health states were assessed using visual analogue scale (VAS) preference and standard gamble (SG) utility data from patients in the US. The ASUI has been included in the Improving Asthma Control Trial (IMPACT), an ongoing, long-term, double-blind parallel group study conducted in the United States (US) and sponsored by the National Institutes of Health (NIH) (S. Sullivan, personal communication).

The objective of the present study was to evaluate ASUI preference weights in Europe to determine whether the multi-attribute utility function, based on preferences derived from a US population, is generalizable across countries. Comparable preferences and derived ASUI algorithms would support the use and validity of the ASUI in multinational clinical trials. As a secondary objective, we derived and evaluated a multi-attribute utility function based on the combined data from the US and Europe.

## Methods

This study was a cross-sectional survey of a sample of patients with asthma. All data were collected by trained interviewers during face-to-face interviews. A total of ninety patients with asthma were recruited from three sites, one in the UK, one in France, and one in Italy. All subjects had to be at least 18 years of age with a diagnosis of asthma. Each site was asked to recruit 10 mild, 10 moderate, and 10 severe patients, as judged by the clinician based on Global Initiative for Asthma (GINA) guidelines for classifying disease severity [10]. In addition, for comparative purposes we included clinical and ASUI data from the original US development study [7].

### Measures

The study subjects completed the Asthma Control Questionnaire (ACQ), the Asthma Quality of Life Questionnaire standardized version (AQLQ-S), and the ASUI. Culturally and linguistically validated [11] UK English, Italian and French translations were available for all the patient reported measures. Subjects also completed a sociodemographic questionnaire with questions on gender, age, education, marital status, comorbidity, and occupational status. The patients' physicians completed a severity of asthma rating, based on the GINA guidelines.

### Asthma Control Questionnaire (ACQ)

The ACQ [12] was used to evaluate control of asthma symptoms. This clinical status scale consists of a composite of asthma symptoms, including awaken at night, waking with symptoms in the morning, shortness of breath, wheeze, limitation in activities, spirometry (completed by clinician), and bronchodilator use. Scores range from 0 to 6, with higher scores indicating more asthma symptoms and related problems.

### Asthma-Symptom Utility Index (ASUI)

The ASUI is an 11-item, preference weighted questionnaire for collecting data on the frequency and severity of four asthma-related symptoms and any side effects of medication therapy [7]. The ASUI measures frequency and severity of cough, wheezing, shortness of breath and sleep disturbance related to asthma. In the ASUI questionnaire, subjects are asked about side effects of asthma medication and the frequency and severity of those they have experienced. The time frame for responses is the past two weeks. Frequency is measured on a four-point scale (i.e., not at all, 1–3 days, 4–7 days and 8–14 days) and severity is measured on a four-point scale (i.e., not applicable, mild, moderate and severe). A single index score is calculated which consists of the preference-weighted individual attribute scores based on a multiplicative multi-attribute utility function derived from a sample of 161 US asthma patients (see Revicki et al. [7] for details). ASUI scores range from 0 to 1.0 with lower scores reflecting greater symptom problems. ASUI scores vary by disease severity and are correlated with Asthma Quality of Life Questionnaire scores [7]. The ASUI was translated and culturally adapted [13] into UK English, Italian and French using an established methodology [13], including 2 forward and 1 backward translations, 3 independent reviews to resolve observed differences in translations, and cognitive interviewing with small samples of asthma patients in each country.

### Asthma Quality of Life Questionnaire (Standardized version)

The Asthma Quality of Life Questionnaire (AQLQ-S) [14-16] was designed as a disease specific measure of HRQL in persons with asthma. It is a self-administered questionnaire that measures symptoms, emotions, environmental stimuli and activity limitation. Scores range from 1 to 7, with higher scores indicating fewer symptoms or better HRQL. Intraclass correlation coefficients range from 0.89 to 0.94 between repeated assessments in stable patients and there is evidence of clinical responsiveness and validity [15-17].

### Developing preference weights

For this study, a combination of VAS and SG tasks were used to generate the multi-attribute preference weighting functions. The health state descriptions and visual props were translated into UK English, Italian and French using standardized translation and cultural adaptation procedures. The VAS and SG scores provide the basic data for deriving the multi-attribute utility functions [7]. The VAS used the Feeling Thermometer as a visual prop [18]. This was a vertical thermometer-shaped scale, 55 cm long, and numerically scaled in units from 0 to 100. The top was labeled "most desirable" and the bottom was labeled "least desirable". For the VAS task, subjects were asked to place the most preferred attribute level or state at 100, the least preferred at 0, and the others in between. Ties were allowed and the relative spacing between pairs of states reflected the subject's judgment about the relative differences in desirability.

The first 5 VAS tasks were used to rate the 5 single-attributes within the ASUI (i.e., cough, wheeze, shortness of breath, sleep disturbance, side effects). For each attribute the patient was given the full set of frequency-severity levels marked on cards, with the best and worst levels explicitly indicated. For each set of ratings, the subject was asked to assume that "all other aspects of your health and abilities are normal". Patients were asked to place the predefined best level at 100 and the predefined worst level at 0 on the Feeling Thermometer. The remaining levels were placed in any order by the patient. The frequency-severity categories for each ASUI attribute were rated separately.

The next VAS task involved rating 10 multi-attribute states. The best and worst health states were placed at 100 and 0, respectively. The patient was given 5 corner states, which include the worst frequency and severity category for one attribute and no problems for the remaining four attributes. The patient was asked to place the 5 corner states on the Feeling Thermometer. Next, they were given the 5 mixed multi-attribute states to place on the Feeling Thermometer. These multi-attribute states varied the severity and frequency of the 5 ASUI attributes (see Table 3). Finally, after preferences for all these states were rated, the patient rated his/her current health.

The SG part of the interview utilized visual props to make the task easier and more understandable for patients [18]. The SG interview required subjects to rate different hypothetical health states based on a gamble between worst asthma state (i.e., frequent and severe symptoms) and best asthma state (i.e., no symptoms) or the certainty of being in the hypothetical health state being measured. In the SG, patients were asked to choose between living for two weeks in the target health state and a gamble. The gamble involved probabilities of either worst asthma state or best asthma state starting with 100% chance of the best state for two weeks and 0% chance of worst asthma state. The probabilities of the best and worst states were then varied until the respondent was indifferent or expressed a dominant choice. To minimize respondent burden, each subject was randomly assigned 1 of 3 sets of health state cards to rate, each set containing 4 of the 10 multi-attribute health states (i.e., corner and multi-symptom states). Finally, all patients rated their current health on the SG.

### Data analysis

The data analyses consisted of five parts: (1) checking item and scale distributions; (2) comparing VAS and SG utility scores across countries; (3) developing the multi-attribute utility functions for the ASUI based on the preference data collected in each country; (4) comparing ASUI scores based on country-specific MAUT functions; and (5) comparing the ASUI scores by clinician-rated asthma severity. The country samples were compared on demographic characteristics, and on mean ACQ score, AQLQ-S scores, and clinician-rated severity measures.

ASUI-US scores were calculated based on the algorithm from the US multi-attribute utility function [7] as follows: ASUI = [1.20 (S1 × S2 × S3 × S4 × S5) - 1.20], where S1 = cough; S2 = wheeze; S3 = dyspnea; S4 = awaken at night; and S5 = side effects. The development of the ASUI preference functions, based on the European data, followed the procedures outlined by Torrance et al. [19] and used by Revicki et al. [7] in developing the US-based ASUI. Preference scores were inspected to identify illogical ratings. The corner and multi-attribute health states were used in a regression analysis, with no intercept, to develop the power function for estimating utilities from VAS scores. We then used these data to determine the multi-attribute value and utility functions based on a multiplicative model [7,20]. The VAS and SG scores for each country were compared using a one-way ANOVA. The ASUI scores were calculated based on the multi-attribute utility functions derived from the data from each of the 3 countries. These ASUI scores, based on the US, UK, French and Italian algorithms, were compared using a one-way ANOVA and intraclass correlation coefficients (ICCs). The Spearman correlation coefficient was used to investigate the relationship between the country-specific algorithm derived ASUI scores and AQLQ-S domain scores and severity scores. The relationship between clinician rated asthma severity and mean ASUI scores, based on the combined (total sample) multi-attribute utility function, were compared using ANOVA.

## Results

A total of 90 subjects completed the study, 30 in each country (Italy, France, UK). All subjects provided consent before participating in the study. Table 1 presents the sociodemographic characteristics of the sample by country. Of the total sample, 56% were female, mean age was 45 years, the majority were living with others (64%), 66% were employed full or part-time, and 46% had a university or post-graduate degree. The percentage of patients with mild (intermittent or persistent), moderate and severe asthma, as defined by clinicians using GINA guidelines, was almost equal (36% mild, 31% moderate, 33% severe). For clinician-rated severity scores, the only significant difference (p < 0.05) was between France and the US. Sixty-three percent to 66% of subjects were rated as having moderate to severe asthma.

**Table 1:** Demographic and Clinical Characteristic of Study Sample

**Characteristic**	**Italy** **N = 30**	**France** **N = 30**	**UK** **N = 30**	**US** **N = 161**
Gender (%)				
Female	50	33	50	59
Male	50	67	50	41
Age in years				
Mean (SD)	48 (12.5)	46 (16.3)	41 (15.1)	34.7 (10.7)
Marital status (%)^1^				
Living alone	10	20	23	
Living with someone	67	70	57	
Other	23	10	20	
Employment status (%)				
Full time paid employment	45	63	47	70
Part time paid employment	10	10	23	7
Unemployed	3	13	3	6
Retired	21	0	13	2
Other	21	13	13	15
Educational attainment (%)				
Elementary school	17	10	0	8
High school graduate	40	20	45	46
College graduate	27	20	52	25
Graduate degree	13	23	3	21
Other (e.g., technical school)	3	27	0	
Co-morbidities (%)^1^				
Arthritis	7	3	17	
Low back pain	27	30	13	
Cancer	3	3	0	
Diabetes	7	7	3	
Heart Disease	10	3	10	
Other	33	23	13	
Physician-rated disease severity (%)				
Mild Intermittent	20	7	13	26
Mild Persistent	13	30	23	32
Moderate	33	30	30	27
Severe	33	33	33	15

^1^Data not collected in the US study

There was very little missing data observed in this study. There were no missing VAS or SG scores, and individual ASUI item scores were missing in 0% to 6.6% of subjects. Individual ACQ scores were incomplete in 0% to 6.6% of subjects, and individual AQLQ-S item scores were missing in 0% to 10% of subjects. However, most subjects had no missing data on the ASUI, ACQ or AQLQ-S.

No significant differences were found between countries on ACQ scores (p > 0.05; see Table 2). Mean AQLQ-S overall and domain scores by country are presented in Table 2. The mean overall AQLQ-S score was 4.73 for Italy, 4.88 for the UK, 5.18 for France, and 5.22 for the US.

**Table 2:** Descriptive Statistics for ACQ and AQLQ-S Scores by Country

**Measure**	**Italy** **Mean (SD)** **N = 30**	**France** **Mean (SD)** **N = 30**	**UK** **Mean (SD)** **N = 30**	**US** **Mean (SD)** **N = 161**
**Asthma Control Questionnaire**				
	1.78 (1.01)	1.65 (1.06)	2.24 (1.14)	1.69 (1.64)*
**Asthma Quality of Life Questionnaire – Standardized Version**				
Symptoms	4.64 (1.30)	5.17 (1.25)	4.57 (1.38)	5.12 (1.24)
Activity	4.91 (1.32)	5.38 (1.17)	5.36 (1.25)	5.43 (1.23)
Emotion	4.68 (1.51)	5.44 (1.36)	4.76 (1.41)	5.11 (1.57)
Environment	4.68 (1.57)	4.73 (1.59)	4.81 (1.33)	5.23 (1.36)
Overall Score	4.73 (1.19)	5.18 (1.19)	4.88 (1.23)	5.22 (1.20)

*Based on severity scale from 0 – 6, not the ACQ

The distributional characteristics of VAS and SG scores by country, including the US were compared. For the corner states (states in which one attribute is described at the worst level and the others are described at the best level), mean VAS scores showed some variability across countries, particularly for medication side effects (range from 0.17 in France to 0.44 in Italy). The ordinal ranking of corner states also varied, though severe dyspnea and severe wheeze were consistently ranked least or second-least desirable for all countries, with the exception of severe wheeze in France. For four out of five multi-symptom states, VAS scores were lowest in the US compared to the other countries. Table 3 presents the ANOVA comparison of VAS preferences for corner and multi-symptom health states. Two corner states (severe awaken at night [p < .05] and severe medication side effects [p < .001]) and all multi-symptom states (p < .001) showed statistically significant differences between countries in mean VAS scores. The source for the difference in severe awaken at night was for the mean comparison between France and the US. Of the European countries, only France and the UK had statistically significant difference in mean VAS scores for any of the multi-symptom health states. VAS preferences were lowest in the US and highest in the UK and Italy.

Similarly, SG scores were lowest in the US versus the European countries for all corner and multi-attribute health states. The ordinal rankings of the multi-symptom states, however, were consistent across all countries. The findings from the ANOVA comparison of SG utilities for corner and multi-symptom health states are summarized in Table 4. Only three health states demonstrated statistically significant differences between countries in mean SG utility scores, severe wheeze (p < 0.001), moderate cough and wheeze (p < 0.001), and moderate cough and dyspnea (p < 0.001). In all cases, the source of these differences were for the mean comparisons between the US and France (p < 0.01 to p < 0.001) and the US and Italy (p < 0.01 to p < 0.001). In general, the US SG utilities were lower than those of Italy, France, and the UK, and the utility scores from France and Italy were comparable. Few substantive differences were seen between the four country groups, given that for 7 of 10 (70%) health states there were no statistically significant differences in mean SG utility scores among countries.

**Table 3:** Comparison of VAS Preferences for Asthma Health States by Country

**State**	**Italy** **Mean (SD)** **N = 30**	**France** **Mean (SD)** **N = 30**	**UK** **Mean (SD)** **N = 30**	**US** **Mean(SD)** **N = 161**	**Overall** **F Value**	**Paired Group** **Comparisons**
**Corner States**^a^						
Severe cough	0.289 (0.245)	0.216 (0.167)	0.246 (0.224)	0.263 (0.254)	0.5	
Severe wheeze	0.212 (0.211)	0.193 (0.130)	0.225 (0.185)	0.239 (0.255)	0.4	
Severe dyspnea	0.125 (0.138)	0.149 (0.116)	0.227 (0.226)	0.158 (0.212)	1.5	
Severe awaken at night	0.252 (0.190)	0.115 (0.143)	0.269 (0.267)	0.255 (0.246)	3.3*	5*
Severe medication side effects	0.439 (0.243)	0.171 (0.199)	0.345 (0.295)	0.253 (0.256)	7.0***	1*** 3**
**Multi-attribute States**^b^						
Moderate cough and dyspnea	0.584 (0.218)	0.488 (0.199)	0.660 (0.215)	0.309 (0.250)	27.1***	3*** 4* 5** 6***
Moderate cough and wheeze	0.443 (0.230)	0.307 (0.159)	0.438 (0.223)	0.219 (0.201)	17.4***	3*** 6***
Severe cough; moderate wheeze and dyspnea	0.296 (0.185)	0.181 (0.118)	0.355 (0.180)	0.172 (0.176)	12.5***	3** 4** 6***
Severe cough; moderate wheeze, and awake at night	0.345 (0.200)	0.251 (0.196)	0.395 (0.195)	0.196 (0.201)	11.4***	3** 6***
Severe cough, dyspnea, and awaken at night; moderate wheeze and side effects	0.112 (0.122)	0.072 (0.068)	0.193 (0.139)	0.075 (0.115)	9.4***	4** 6***

Pairwise comparisons between means were performed using Scheffe's test of multiple comparisons.

1 = Italy vs. France, 2 = Italy vs. UK, 3 = Italy vs. US, 4 = France vs. UK, 5 = France vs. US, 6 = UK vs. US.

***<.001, ** <.01, *<.05.

^a ^For corner states, one symptom is described as severe and frequent while other remaining symptoms reflect no problem.

^b ^The multi-attribute states vary symptom severity as listed, with other remaining symptoms reflecting mild severity. Frequency remains constant for each symptom within each health state.

**Table 4:** Comparison of SG Utilities for Asthma Health States by Country

**State**	**Italy** **Mean(SD)** **N = 30**	**France** **Mean(SD)** **N = 30**	**UK** **Mean(SD)** **N = 30**	**US** **Mean(SD)** **N = 161**	**Overall** **F Value**	**Paired Group** **Comparisons**
**Corner States **^a^						
Severe cough	0.795 (0.121)	0.850 (0.176)	0.755 (0.148)	0.689 (0.213)	2.5	
Severe wheeze	0.870 (0.103)	0.880 (0.067)	0.765 (0.172)	0.661 (0.208)	7.3***	3* 5*
Severe dyspnea	0.720 (0.236)	0.760 (0.191)	0.710 (0.165)	0.602 (0.246)	2.0	
Severe awaken at night	0.860 (0.145)	0.750 (0.176)	0.780 (0.157)	0.667 (0.247)	2.6	
Severe medication side effects	0.850 (0.189)	0.775 (0.118)	0.795 (0.174)	0.662 (0.230)	3.3*	
**Multi-symptom States**^b^						
Moderate cough and dyspnea	0.835 (0.173)	0.835 (0.099)	0.715 (0.198)	0.674 (0.234)	5.1**	3* 5*
Moderate cough and wheeze	0.778 (0.180)	0.780 (0.130)	0.675 (0.168)	0.600 (0.241)	5.9***	3* 5*
Severe cough moderate wheeze and dyspnea	0.730 (0.193)	0.800 (0.118)	0.755 (0.146)	0.615 (0.212)	3.8*	
Severe cough; moderate wheeze, dyspnea, and awake at night	0.720 (0.250)	0.760 (0.120)	0.675 (0.177)	0.589 (0.243)	2.6	
Severe cough, dyspnea, and awaken at night; moderate wheeze and side effects	0.640 (0.313)	0.630 (0.210)	0.665 (0.189)	0.455 (0.275)	3.3*	

Pairwise comparisons between means were performed using Scheffe's test of multiple comparisons.

1 = Italy vs. France, 2 = Italy vs. UK, 3 = Italy vs. US, 4 = France vs. UK, 5 = France vs. US, 6 = UK vs. US.

***<.001,**<.01,*<.05.

^a ^For corner states, one symptom is described as severe and frequent while other remaining symptoms reflect no problem.

^b ^The multi-attribute states vary symptom severity as listed, with other remaining symptoms reflecting mild severity. Frequency remains constant for each symptom within each health state.

We attempted to fit similar multi-attribute utility function models as those determined from the earlier US study data to the data from Italy, France, and the UK. For the UK, a multiplicative multi-attribute utility function was acceptable and was fit to these data. For France and Italy, there was no support for the multiplicative function, and additive function models were fit. Given these observed differences in deriving ASUI weighing algorithms, we also determined the best model (i.e., additive or multiplicative) for the combined US, UK, French, and Italian data. For the combined data, we were able to fit a multiplicative multi-attribute utility function and derived ASUI scores based on this algorithm. Therefore, in this study we derived 5 different ASUI scores based on the US (ASUI-US), UK (ASUI-UK), French (ASUI-FR), Italian (ASUI-IT), and combined sample algorithms (ASUI-ALL).

Mean ASUI scores for each sample were calculated using each country-specific scoring algorithm and are provided in Table 5. Mean ASUI scores were lowest for all samples when calculated using the US scoring algorithm, with mean scores ranging from 0.63 for the UK sample to 0.77 for both the French and Italian samples. Mean scores ranged from 0.86 (UK) to 0.93 (US) using the Italian algorithm, 0.88 (UK) to 0.95 (US) using the French algorithm, and 0.86 (UK) to 0.93 (US, Italy) using the UK algorithm. Using the combined sample algorithm, the mean score for the UK sample was 0.76 and for the US, French, and Italian samples was 0.86.

**Table 5:** Descriptive Statistics and Distributional Characteristics of ASUI Scores using Country-Specific Algorithms

**Sample**	**Mean (SD)**	**Median**	**Range** **(Min/Max)**
**US Algorithm**			
US	0.76 (0.19)	0.78	0.08–1.00
France	0.77 (0.22)	0.84	0.30–1.00
Italy	0.77 (0.20)	0.82	0.13–1.00
UK	0.63 (0.23)	0.63	0.19–1.00
**Italian Algorithm**			
US	0.93 (0.12)	0.97	0.18–1.00
France	0.92 (0.13)	0.97	0.54–1.00
Italy	0.92 (0.17)	0.99	0.17–1.00
UK	0.86 (0.16)	0.91	0.38–1.00
**French Algorithm**			
US	0.95 (0.11)	0.99	0.13–1.00
France	0.94 (0.11)	0.99	0.66–1.00
Italy	0.94 (0.15)	1.00	0.23–1.00
UK	0.88 (0.15)	0.94	0.42–1.00
**UK Algorithm**			
US	0.93 (0.09)	0.96	0.45–1.00
France	0.92 (0.10)	0.98	0.66–1.00
Italy	0.93 (0.11)	0.97	0.48–1.00
UK	0.86 (0.12)	0.89	0.55–1.00
**Combined Algorithm**			
US	0.86 (0.14)	0.90	0.16–1.00
France	0.86 (0.17)	0.93	0.45–1.00
Italy	0.86 (0.17)	0.91	0.21–1.00
UK	0.76 (0.19)	0.79	0.30–1.00

Table 6 presents mean ASUI scores for the total sample (US, UK, Italy, and France) as calculated using each country-specific algorithm and the combined sample algorithm. The mean was lowest using the US algorithm (0.75) and highest using the French algorithm (0.94). Pairwise comparisons between means were performed using Scheffe's test of multiple comparisons. Statistically significant (p < .001) differences in mean ASUI scores were found for 7 of 10 paired comparisons of country-specific algorithms. Mean scores using the Italian, French, and UK algorithms (Italy vs. France, Italy vs. UK, France vs. UK) were *not *significantly different. The ASUI scores based on the country-specific algorithms were correlated from 0.971 to 0.995 (p < 0.0001). The ICCs comparing the country-specific ASUI scores ranged from 0.44 (ASUI-US and ASUI-FR) to 0.97 (ASUI-FR versus ASUI-IT), with 70% of ICCs greater than 0.74.

**Table 6:** Descriptive Statistics and Distributional Characteristics of ASUI Scores by Country Algorithm in Total Sample**

**Algorithm**	**Mean (SD)**	**Median**	**Range** **(Min/Max)**	Floor (%)*	Ceiling (%)*
US	0.75 (0.20)	0.78	(0.08–1.00)	0.41	12.60
France	0.94 (0.12)	0.99	(0.13–1.00)	0.41	14.63
Italy	0.92 (0.13)	0.97	(0.17–1.00)	0.41	12.60
UK	0.92 (0.10)	0.96	(0.45–1.00)	0.41	12.60
All	0.85 (0.16)	0.90	(0.16–1.00)	0.41	12.60

* Floor = percent who answered minimum value; Ceiling = percent who answered maximum value

** All pairwise comparisons between means were statistically significant at p < .0001 except Italy vs. UK (p = 0.9234).

**Figure 1:**
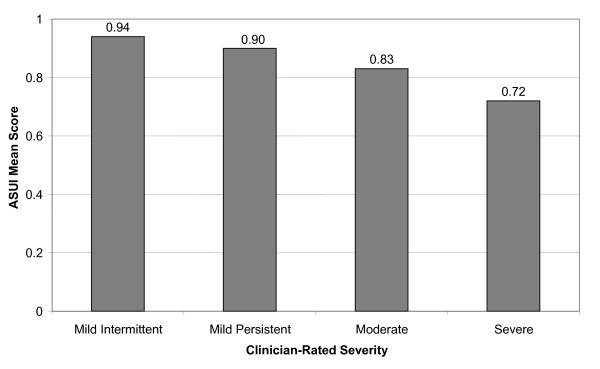
Mean ASUI Scores (Combined Algorithm) by Clinician-Rated Disease Severity: Total Sample (UK, Italy, France, US).

Using the combined algorithm for the total sample, mean ASUI scores decreased with increased asthma severity, as rated by the clinician (mild intermittent – 0.94, mild persistent – 0.90, moderate – 0.83, severe – 0.72) (p < 0.0001; see Figure 1).

Spearman correlations between each country-specific algorithm based ASUI scores and the AQLQ-S domain and overall, ACQ and clinician-rated severity scores were calculated (Table 7). Correlations between ASUI scores and AQLQ-S domain and ACQ scores were generally moderate to high and statistically significant (p < 0.0001) regardless of algorithm. Correlations between ASUI scores and clinician-rated severity scores were generally not as high as those between ASUI and AQLQ-S and ACQ scores. Most importantly, the magnitude and direction of correlations between the ACQ and clinician-rated severity measures and the various ASUI scores were comparable. The relationship between the AQLQ-S scores and the ASUI scores generated by the country-specific algorithms were also comparable.

**Table 7:** Spearman Correlations Between ASUI and AQLQ-S Domain and Overall Scores, Adequacy of Asthma Control (ACQ), and Clinician-Rated Severity of Disease

	**ASUI** **Country-specific Algorithm**				
	
	**ASUI-US**	**ASUI-UK**	**ASUI-FR**	**ASUI-IT**	**ASUI-ALL**
**Asthma Quality of Life Questionnaire**					
Symptom	0.829	0.827	0.812	0.814	0.829
Activity limitation	0.624	0.632	0.639	0.637	0.635
Emotional function	0.609	0.596	0.589	0.589	0.606
Environmental stimuli	0.551	0.570	0.564	0.565	0.564
Overall Score	0.733	0.735	0.729	0.729	0.738
**Asthma Control Questionnaire**	-0.610	-0.616	-0.594	-0.589	-0.611
**Clinician-rated Severity**	-0.496	-0.494	-0.485	-0.478	-0.501

*All combinations are significant at p < .0001

## Discussion

This study evaluated whether the preferences and utilities for asthma symptom-related health states were comparable across a sample of asthma patients from the US and selected European countries. In addition, we evaluated the multi-attribute utility functions derived from these country-specific preference/utility data. We found evidence that asthma patients in different countries rate the same symptom-defined health states somewhat differently. Although there were few differences in the SG utilities for the corner and multiple symptom states, we did observe differences on the severe wheeze corner state and the moderate level multi-symptom states (involving cough and wheeze and cough and dyspnea). In all cases, the differences were mainly between the US and Italian sample and between the US and French sample utility estimates. The US subjects tended to rate these health states as worse than the Italian and French subjects. It is likely that cultural differences in perception and valuation of some asthma symptoms may exist, and that these differences were expressed between the French and Italian subjects and those from the US. It is interesting to note that the UK subjects reported utilities that were between those reported by the US and the French and Italian samples.

As expected, there were differences between mean VAS preference and SG utility scores for the multi-attribute asthma states. Differences of this magnitude have been observed in previous studies [8,21-23]. The differences observed are likely due to the differences in the VAS and SG methods for collecting preferences; for example, the SG method introduces risk into the assessment of utilities. In addition, the SG utilities were derived using a 2 week time period which was done to capture the variations in symptom experience for patients with asthma. This was the identical approach taken in the U.S. study [7]. Longer time periods for the SG exercise might have resulted in different preference scores.

The generated multi-attribute utility functions for the ASUI differed between the different countries. Based on the US and the UK data, a multiplicative multi-attribute utility function was fit to the utility data, while the French and Italian data supported an additive model. The resultant ASUI scores were significantly higher for the UK, French, and Italian based algorithms compared with the US algorithm. The combined data algorithm was based on a multiplicative multi-attribute utility function, and the resultant mean ASUI scores differed significantly from the US based, UK based, Italian based, and French based ASUI scores. Clearly, there are differences in mean ASUI scores among the different preference weighting algorithms. Based on these data, the US derived algorithm may not fit the preference structure of asthma patients from France or Italy.

We examined the correlations among the different country-specific algorithm derived ASUI scores and found significant correlations among the different scores. The strength of these correlations suggest that although the distribution of the different ASUI scores may be shifted toward lower or higher scores, the relative rank ordering of mean scores in patients with asthma symptoms are maintained. This is further supported by the relationships observed between the 5 different ASUI scores and the AQLQ-S scores, the ACQ and clinician-rated disease severity. The observed results for the total sample indicate very comparable correlations between the different ASUI scores and the asthma-specific quality of life scores. For example, AQLQ-S symptom scores were correlated from 0.81 (for ASUI-FR or ASUI-IT) to 0.83 (for ASUI-US or ASUI-ALL) with the different ASUI scores, and larger correlations were seen between ASUI scores and AQLQ-S symptom scores than for environmental stimuli, activity limitation, and emotional function scores. More importantly, comparable magnitude correlations were seen between the ASUI scores and clinician ratings of asthma severity. When the mean ASUI score from the combined sample algorithm is compared by physician-rated asthma severity groups, we observe that patients with severe persistent asthma have ASUI scores that are significantly lower than those with less severe asthma severity. These findings are consistent with those reported in the original ASUI development study [7].

The findings of this study should be interpreted in light of several study limitations. First, the measures of disease severity differed somewhat between the European and US samples. The clinician-rated severity for the European study was based on GINA guidelines, while asthma severity for the US study was based on physician global assessment of severity from mild to severe. Second, the VAS preference and SG utility interviews were completed for all health states in the US sample, but in only a sub-sample of subjects in Europe. There were fewer available data on which to base mean SG utilities in Europe and this may have resulted in somewhat unstable utilities for the health states. Finally, the sample sizes by country for Europe were 30 each, compared with 161 in the US sample. Given the relatively small samples, one or two respondent preference ratings, based on different clinical characteristics, could potentially skew the findings. Additional research is needed to confirm these utility and preference estimates in the European samples.

## Conclusion

In summary, the results of this study indicate that preferences for asthma-related symptoms and multiple symptom states differ between France and Italy and the UK and the US. Because of these differences, the multi-attribute utility functions derived within countries were somewhat different. Despite these differences, the results indicate that each of the derived algorithms captures a similar rank ordering of patients by disease severity, although the ASUI score distributions may be shifted somewhat. Therefore, as long as the same algorithm is used within an international clinical trial, the relative ordering of mean ASUI scores by disease severity is preserved. The greater range of ASUI scores, based on the US or combined algorithm, suggests that either of these two algorithms may be more responsive to changes in clinical status within clinical trials. However, data on the responsiveness of the ASUI scores requires further research. The ASUI represents a useful and valid measure of preference-weighted asthma symptoms for use in clinical trials and clinical management. The findings of this study suggest that the ASUI may be a complementary patient-reported outcome for clinical studies and may be useful for applications in cost-effectiveness studies comparing different asthma treatments.

## Abbreviations

ACQ Asthma Control Questionnaire

AQLQ-S Asthma Quality of Life Questionnaire

ASUI Asthma-Specific Utility Index

GINA Global Initiative for Asthma

HRQL Health-Related Quality of Life

IMPACT Improving Asthma Control Trial

NIH National Institutes of Health

SG Standard Gamble Utility

VAS Visual Analogue Scale

## Competing interests

Ann-Christin Mörk was an employee of AstraZeneca at the time this study was conducted. The remaining authors declare that they have no competing interests.

## Authors' contributions

Emuella Flood drafted the manuscript and participated in the design, data collection, and data analysis. Dennis Revicki helped draft the manuscript and participated in the design, data analysis and interpretation of the findings. Erwin De Cock reviewed the manuscript and assisted in data collection and data analysis. Ann-Christin Mörk reviewed the manuscript and participated in the design and implementation of the study. All authors read and approved the final manuscript.
